# CypA, a Gene Downstream of HIF-1α, Promotes the Development of PDAC

**DOI:** 10.1371/journal.pone.0092824

**Published:** 2014-03-24

**Authors:** Huan Zhang, Jing Chen, Fenghua Liu, Chuntao Gao, Xiuchao Wang, Tiansuo Zhao, Jingcheng Liu, Song Gao, Xiao Zhao, He Ren, Jihui Hao

**Affiliations:** National Clinical Research Center for Cancer, Key Laboratory of Cancer Prevention and Therapy, Department of Pancreatic Cancer, Tianjin Medical University Cancer Institute and Hospital, Tianjin, China; University of Nebraska Medical Center, United States of America

## Abstract

Hypoxia-inducible factor-1α (HIF-1α) is a highly important transcription factor involved in cell metabolism. HIF-1α promotes glycolysis and inhibits of mitochondrial respiration in pancreatic ductal adenocarcinoma (PDAC). In response to tumor hypoxia, cyclophilin A (CypA) is over-expressed in various cancer types, and is associated with cell apoptosis, tumor invasion, metastasis, and chemoresistance in PDAC. In this study, we showed that both HIF-1α and CypA expression were significantly associated with lymph node metastasis and tumor stage. The expression of CypA was correlated with HIF-1α. Moreover, the mRNA and protein expression of CypA markedly decreased or increased following the suppression or over-expression of HIF-1α in vitro. Chromatin immunoprecipitation analysis showed that HIF-1α could directly bind to the hypoxia response element (HRE) in the CypA promoter regions and regulated CypA expression. Consistent with other studies, HIF-1α and CypA promoted PDAC cell proliferation and invasion, and suppressed apoptosis in vitro. Furthermore, we proved the combination effect of 2-methoxyestradiol and cyclosporin A both in vitro and in vivo. These results suggested that,CypA, a gene downstream of HIF-1α, could promote the development of PDAC. Thus, CypA might serve as a potential therapeutic target for PDAC.

## Introduction

Pancreatic cancer is highly malignant with poor prognosis [1]. In China, it is the fifth common cause of death in cancer [2]. Pancreatic cancer typically spreads rapidly and is seldomly detected at its early stages, which mainly explains its morbidity. Signs and symptoms usually do not appear until pancreatic cancer is quite advanced and surgical removal is no longer possible. Moreover, no effective treatment exists for eliminating residual cancer cells after surgery, which is still the major form of treating pancreatic cancer [3]. Therefore, understanding the molecular basis of the disease is highly desirable for developing new strategies for prevention and treatment of pancreatic cancer.

HIF-1α is an important transcription factor that renders tumor cells to adapt to hypoxia. It has important functions in cancerous transformation, chemoradiotherapy resistance, and tumor progression [4]–[9]. HIF-1α regulates the expression of many genes, including erythropoietin, VEGF, heme oxygenase-1, enolase, lactate dehydrogenase A, and aldolase [6]–[9]. Tumor cells can also increase the expression of HIF-1α by activating AKT under normoxia, and activate downstream target genes under normoxia [7]–[9]. A recent study reported that HIF-1α had a higher expression in pancreatic cancer than that in the normal tissue, and that the level of HIF-1α protein expression is related to clinical stage and lymph node metastasis [10]. Thus, HIF-1α has a potential value to be a new therapeutic target for pancreatic cancer. Targeted therapy against HIF-1αexpression in PDAC has been investigated recently [4]–[11]. 2-Methoxyestradiol (2ME) single-agent therapy has limited response because of its low bioavailability [10], [11], whereas combination therapy exhibits promising in vitro results, which warrant further in vivo investigations [12].

In response to tumor hypoxia, CypA is also involved in the process of cell apoptosis [13]. CypA is over-expressed in various cancer types and is associated with tumor invasion, metastasis, and chemo-resistance [14]–[17]. Recent reports indicated that the inhibition of the function of CypA reduced the malignant potential of pancreatic cancer cells both in vitro and in vivo [18]. Cyclosporin A (CsA), an inhibitor of CypA, induces apoptosis in various cancer cells by inhibiting peptidyl-prolyl cis-trans isomerase (PPIase) activity of CypA [15]. However, its immunosuppressive effects limit the application. Preliminary studies have revealed that CsA can be administered in tolerable and manageable doses in the combination therapy of various cancer types [19], [20]. However, the application of CypA in PDAC is rarely reported.

In this study, we investigated the prognostic value of HIF-1α and CypA protein expression in primary PDAC tissues, and discovered the correlation between them. Moreover, we investigated the biological effects of CypA and HIF-1α on PDAC in vitro, as well as the combination effect of 2ME and CsA both in vitro and in vivo.

## Materials and Methods

### 1. Ethics statement

The use of human samples in this study was approved by the Ethics Committee of Tianjin Cancer Hospital.The research involving human participants and animal experiments had been approved by our hospital and our equivalent committee. The participants provided their written informed consents to participate in this study, but could not be provided because of too many written informed consents in Chinese or it would not be feasible for me to provide all informed consent because of their number and non-English language.

All animal work had been conducted according to relevant national and international guidelines.

This study was carried out in strict accordance with the recommendations in the Guide for the Care and Use of Laboratory Animals of the National Institutes of Health (Tianjin cancer hospital). The protocol was approved by the Committee on the Ethics of Animal Experiments of the Tianjin cancer hospital. All surgery was performed under sodium pentobarbital anesthesia, and all efforts were made to minimize suffering.

### 2. Patients and tissue specimens

Pancreatic tumor samples were obtained from patients undergoing surgical resection at Tianjin Cancer Institute and Hospital. Normal pancreatic tissues were obtained from patients undergoing surgical resection for diseases other than pancreatic cancer. The usage of human samples in this study was approved by the Ethics Committee of Tianjin Cancer Hospital. The specimens were obtained from 95 patients (ages, 36 to 79 years) with histological diagnosis of PDAC between July 2003 and April 2009. In brief, diagnosis were made base on the cellular features by light microscopy, immunohistochemical (IHC) stainings, and clinical observations (including the clinical symptoms, tumor stage, lymph node metastasis and CA19-9 value). Patients' clinical characteristics and pathological features were shown in Table 1.

**Table 1:** Association between HIF-1α, CypA expression levels and the clinical factors.

	HIF-1α		P	CypA		P
	−/+/++	+++		-	+	
1.Age						
≤55	70	19	0.358	35	54	0.717
>55	49	20		30	39	
2.Sex						
man	69	21	0.789	41	49	0.257
woman	50	18		24	44	
3.Tumor stage						
T2	59	11	0.032*	55	15	<0.001*
T3	60	28		10	78	
4.Lymph node metastasis						
N0	69	32	0.012*	50	51	0.007*
N1	50	7		15	42	
5.CA19-9 (kU/L)						
≤500	79	21	0.223	35	65	0.059
>500	40	18		30	28	

### 3. Immunohistochemistry (IHC) of HIF-1α and CypA

Paraffin-embedded pancreatic tissue sections were cut, deparaffinized, and rehydrated with xylene and graded alcohols. Antigen retrieval was carried out in 5 mM citrate buffer. After the inactivation of endogenous peroxidase with 3% H_2_O_2_, the sections were blocked with goat serum and incubated with anti-HIF-1α (Santa Cruz Biotechnology, 1∶100 dilution, overnight at 4°C) or anti-CypA (Abcam, 1∶350 dilution, overnight at 4°C) antibodies. The sections were then incubated with biotinylated secondary antibody and streptavidin-biotin-peroxidase. Diaminobenzidine was used as a chromogen substrate. Finally, the sections were counterstained with hematoxylin. Two independent observers particularly experienced in IHC evaluated the slides; all cases with discrepant interpretations were discussed using a double-headed microscope prior to reaching a consensus. The immunoreactivity of HIF-1α was scored as +, ++, +++, and ++++ as previously described [21]. To determine CypA expression, the percentage of positive cells and staining intensity points were added to produce a weighted score for each case. The percentage of positive cells was categorized as: 1 point, no more than 10% positive cells; 2 points, 11% to 50% positive cells; 3 points, 51% to 80% positive cells; and 4 points, > 81% positive cells. The staining intensity was rated as 0, 1, 2, or 3 points for no staining, weak, moderate, and strong intensity staining, respectively. Specimens were classified into the following four groups according to their weighted score: -, 1 to 2 points; +, 3 points; ++, 4 to 5 points, and +++, 6 to 7 points. For statistical purposes, specimens were classified into the following two groups: (−) and (+), low reactivity group; (++) and (+++), high reactivity group, which were enrolled into negative (−) and positive (+) categories.

### 4. Cell culture and treatments

#### Cell culture

Human pancreatic carcinoma cell line Mia-paca-2 and BXPC3 were obtained from the American Type Culture Collection A (Manassas, VA, USA). All cells were maintained in Dulbecco's modified eagle's medium supplemented with 10% fetal bovine serum (FBS), 100 IU/mL penicillin, 100 μg/mL streptomycin, and 2 mmol/L glutamine. Cells were grown at 37°C in a humidified atmosphere of 95% air and 5% CO_2_.

Divided groups: the cells were divided into 4 groups namely as control group, siRNA HIF-1α (section 4: 2ME), siRNA CypA (section 4: CsA), and siRNA HIF-1α+siRNA CypA (section 4: 2ME+CsA) groups.

#### siRNA duplexes and transfection

Two HIF-1α siRNA duplexes (Ribobio, Guangzhou, China), two pcDNA HIF-1α duplexes (Ribobio, Guangzhou, China), and two CypA siRNA plasmids (GenePharma, Shanghai, China) were devised. The knockdown efficiency was assessed by western blot analysis (data not shown). The most efficient duplexes for suppression were as follows: siRNA-HIF-1α forward: 5′-CUGAUGACCAGCAACUUGAdUdU-3′, reverse: 5′-dTdTGACUACUGGUCGUUGAACUS-3′; and siRNA-CypA forward: 5′-GGGAGGCCAGGCTCGTGC-3′, reverse: 5′-GCACGAGCCTGGCCTCCC-3′. The most efficient duplexes for over-expression of HIF-1α were the domains of over-expressed HIF-1α. Transfection of cells with siRNAs was carried out using Lipofect AMINETM 2000 (Invitrogen, Carlsbad, CA, USA) according to the manufacturer's instructions. RNA and protein were extracted from the transfected cells.

#### Real-time polymerase chain reaction (RT-PCR) assays

Total RNA was extracted by Trizol (Invitrogen, Carlsbad, CA, USA) according to the manufacturer's instructions. cDNA was synthesized using a first-strand cDNA synthesis kit (TaKaRa, Dalian, China). Each sample was prepared in triplicate. The mean values were used for calculation. β-actin was used as the loading control. The comparative detailed ΔCt was used to analyze the results. All data were normalized against ΔCt of β-actin for each sample to obtain a relative level of gene expression. Forward and reverse primers were as follows: 5′-GCAAGCCCTGAAAGCG-3′ and 5′-GGCTGTCCGACTTTGA-3′ (HIF-1α); 5′-AGCTACTGCCATCCAATCG-3′ and 5′-TGTGCTGGCCTTGGTGAG-3′ (VEGF); 5′-CCTGGGCATGGAGTCCTGTG-3′ and 5′-AGGGGCCGGACTCGTCATAC-3′ (β-actin); and 5′-CAAGGTCCCAAAGACAGCAGA-3′ and 5′-AAGATGCCAGGACCCGTATGC-3′ (CypA).

#### Western blot analysis

Cells were rinsed by ice-cold PBS and lysed with lysis buffer (20 mM Tris-HCl, pH 6.8, 150 mM NaCl, 1 mM EDTA, 0.2% sodium dodecyl sulfate (SDS), 1 mM b-glycerophosphate, 2 mM phenylmethylsulfonyl fluoride, 10 μg/mL leupeptin, 50 mmol/L NaF, and 1 mmol/L Na_3_VO_4_). Cell extracts (20 μg protein per lane) were separated by SDS–polyacrylamide gel electrophoresis and transferred to polyvinylidene fluoride membranes (Millipore Corp, Bedford, MA, USA). The membranes were blocked with blocking buffer and incubated with the primary antibody at 4°C on the table concentrator overnight. The membranes were washed and brooded on the secondary horseradish peroxidase-labeled antibody. Bands were visualized with enhanced chemiluminescence (Thermo, Biotechnology, Germany). The requested antibodies were as follows: HIF-1α, VEGF (Santa Cruz Biotechnology, Santa Cruz, CA, USA), β-actin, and CypA (Abcam, USA). Western blot lanes were analyzed by Image J Software using reflectance densitometry.

#### Chromatin immunoprecipitation (ChIP) assays

A commercial chip assay kit (Upstate Biotechnology, Waltham, USA) was used following the manufacturer's instructions. After treatment, each sample group was incubated with 1% formaldehyde to cross-link the DNA–protein complexes. After washing with ice-cold PBS for three times, cells were lysed in SDS lysis buffer. The lysate was sonicated to shear DNA into 200 bp to 1000 bp fragments. Anti-HIF-1α antibodies (Santa Cruz Biotechnology, Santa Cruz, USA) were then used to immunoprecipitate the cross-link protein (HIF-1α) at 4°C overnight, and IgG was used as the negative control. After washing, centrifugation, elution, and reversal, the DNA was recovered with a phenol/chloroform/isoamyl mixture and precipitated by alcohol. The DNA was used as a template for RT–PCR of the CypA and VEGF binding sites (positive control). The sequences of the VEGF primers were previously described. The sequences of the CypA primers were as follows: CypA-1 forward: 5′-AGTCCCATGCCGCAGCCACC-3′, reverse:5′-CCTCCCGCCCCTTTTATACCAC-3′; CypA-2 forward: 5′-GGCCTGCGTTCGCCTCAGTT-3′, reverse:5′-GTGGCTGCGGCATGGGACTC-3′; and CypA-3 forward: 5′-ACCACCATGCCCAGCTAATT-3′, reverse: 5′- TGATCGCATCACTGCATTCC-3′. The sequences of the negative primers were as follows(not including HRE): forward: 5′-GCTGCTGCACCTGGACGTTG-3′, reverse:5′-CGGCTCACTGCAACCTCCCC-3′; The PCR products were separated by 1% agarose. VEGF was used as the positive control in the course of the experiment.

#### Luciferase assay

5×10^4^ cells per well in 12-well plates were cultured without antibiotics overnight and then transfected with CypA-promotor (we used VEGF-promotor as positive control and CypA-promotor-mutation as negative control) and pcDNA3.1-HIF-1α. After 24 h, cells were washed with phosphate-buffered saline (PBS), subjected lysis, and their luciferase activities were measured by using a dual luciferase assay kit (Promega). The results were normalized against Renella luciferase. All transfections were performed in triplicate. (HIF-1α primers forward: TGC TCTAGATCTCTAGTCTCACGAGGGGTTTCC; Reverse: CGCGGATCC GATGCTACTGCAATGCAATGGTT; CypA primers forward: CGGGGTACCGTCTCACCACAACCCTATCCTGGTT; reverse: CTAGCTAGCGGCCTCCCGCCCCTTTTATA; GCGTG mutated into GCATG).

#### MTT assays

MTT was used to observe and compare the cell proliferation ability. The cells were divided into three groups, namely, control, transfected siRNA-HIF-1α, or siRNA-CypA. In brief, 2×10^3^ cells in 200 μL of culture medium were plated into each well of a 96-well plate. After culturing cells for an appropriate time, 10 μL of 5 mg/mL MTT was added into each well. Cells were then cultured for 4 h. The cell culture medium was replaced with 100 μL of dimethyl sulfoxide (DMSO). Thirty minutes after the addition of DMSO, the plates were placed on a microplate autoreader (Bio-Rad, Hercules). Optical density (OD) was read at a wavelength of 570 nm, and cell growth curves were determined according to the OD value.

#### Cell apoptosis assays

Cells were dosed 24 h after plating, and divided into three groups, namely, control, transfected siRNA-HIF-1α, and siRNA-CypA. After treatment, cells were tested according to the protocol of Biolegend kit (Cat No. 640906, Biolegend, USA). In brief, cells were washed with PBS and resuspended in Annexin V binding buffer at a concentration of 10^6^ cells/mL. After transferring a 100 μL cell suspension to a 5 mL test tube, 5 μL of FITC Annexin V and 5 μL of PI solution were added to the cell suspension. After 15 min of incubation, 400 μL of Annexin V Binding Buffer was added to each tube, and apoptosis was analyzed by flow cytometry.

#### Cell scratch assays

The cells and transfectants were seeded in six-well plates and cultured for 72 h to obtain 80% confluent monolayer. A scratch was created by scraping the cells using a plastic pipette tip, and the medium was replaced with a fresh medium. Images were captured immediately at 0, 6, 12, and 24 h.

#### Cell invation assays

The 8 μm pore polycarbonate membrane transwell chambers (Costar) were used, and 1×10^5^ cells were cultured in the upper chamber with serum-free medium. The lower chamber contained complete medium (10% FBS). After incubation at 37°C and 5% CO_2_ for 12 h, the cells that adhered to the top surface of the membrane were removed with a cotton applicator, whereas the cells that migrated to the bottom surface were fixed with 70% methanol and stained with crystal violet. The migrating cells on the bottom surface of the membrane were photographed and counted using an inverted microscope. After staining with crystal violet, OD values were detected.

### 5. Use of drugs to inhibit HIF-1α and/or CypA

The dose-dependent effects of 2ME and CsA were determined as single agents on the growth of Mia-paca-2 or BXPC3 cells. Cells were seeded on a 96-well culture plate, and dosed with different concentrations of 2ME or CsA. Cells were treated with 0, 50, 100, and 200 μM 2ME with 0, 1, 2, and 4 μM CsA. The cells were dosed 24 h after plating with gradient 2ME or CsA to determine the IC50 and dose-dependent response of both agents. The IC50 values of 2ME and CsA were used in designing experiments to determine the synergistic anti-proliferative interaction between these compounds. For combination studies, the ratio of the two drugs was established from the IC50 values for the inhibition of cell proliferation. Five doses of agents, ranging from 10 μM to 50 μM 2ME and 330 nM to 1670 nM CsA (combination ratio of 5∶1, 2∶1, 1∶1, 1∶2, and 1∶5, respectively), were used for the combination studies. Cells were harvested after 48 h of treatment. The possible synergistic growth inhibitory interactions between 2ME and CsA were examined by using isobolographic analysis [22]–[26]. The three possibilities, namely, γ<1, γ = 1, and γ>1, indicated synergy, additive effect, and antagonism, respectively. The interaction index values ranged from 0.2 to 0.5, which indicated the strong synergism in the growth inhibitory effect of 2ME/CsA (data not shown).

MTT assays, cell apoptosis assays, cell scratch assays and cell invation assays were shown in section 3.

### 6. Animal experiments

BXPC3 cells (2×10^7^) were injected subcutaneously into the right flanks of female severe combined immunodeficiency (SCID) mice. After three weeks post-injection, the mice were randomized into four treatment groups (n = 3 for each group), namely, control, 2ME, CsA, and 2ME+CsA groups. The treatment schedule started at day 0 with the intra-abdominal injection of CsA (10 mg/kg per day for 5 d) or 2ME (50 mg/kg per day for 14 d) for single-agent therapy, and half dose of 2ME/CsA (25 mg/kg for 2ME and 5 mg/kg for CsA) for combination therapy. The control group received PBS at day 0 and then every day for a total of 14 d in a volume of 100 μL. The treatment schedule was repeated every third week for a total of six weeks. The physical condition of the animals, including fur-roughing, shedding and local trauma at the site of injection, and decrements in general animal activity, were regularly monitored. Tumor volume was measured every 10 d using a Vernier caliper, and calculated using the following formula: V = π/6× length × width^2^. Blood samples were collected from the eyes at the end of the sixth week of treatment for counting and analysis of liver and renal function. The mice were sacrificed immediately after the blood samples were obtained, and the tumor samples were then isolated from the mice and photographed. All tumor samples were paraffin-embedded for IHC analysis as detailed above in section 2.

### 7. Statistical analysis

All the clinical characteristics of patients were compared by the χ^2^ test for categorical variables. Student's t-test or ANOVA test for unpaired data was used to compare mean values. Spearman's rank correlation coefficient test was conducted to test association among ordinal variables. All probability values were two sided. Analyses were performed using SPSS13.0 statistical analysis software. Values are presented as median (minimum-maximum) or mean ± SD, unless otherwise stated. P<0.05 was considered statistically significant for all tests.

## Results

### 1. CypA and HIF-1α are over-expressed in PDAC and their expression levels predict poor outcome

**Figure 1:**
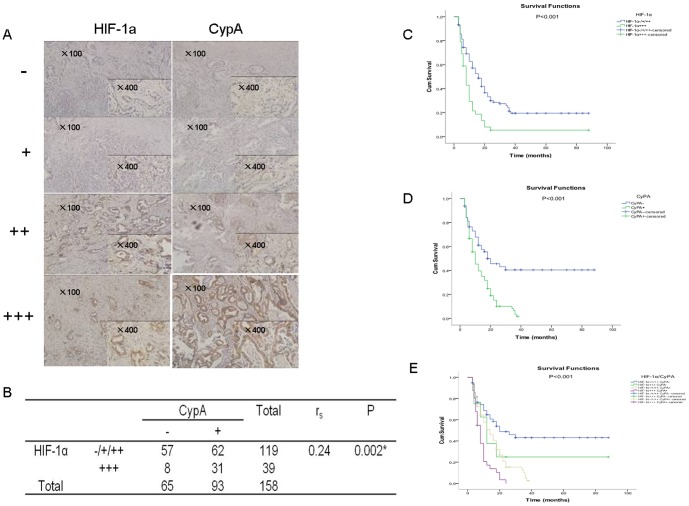
Validation of expression of HIF-lα and CypA in PDAC tissues and analysis of the clinical significance. **A.** The IHC results of HIF-lα and CypA. CypA protein expression was significantly correlated with HIF-lα, as detected by immunohistochemical staining on PDAC. Left panels: various expression levels of HIF-lα protein. Right panels: expression of CypA protein of the same samples in the adjacent section. **B.** Cross tabulation of HIF-lα and CypA expression levels in 158 cases of PDAC. Expression of CypA was correlated with HIF-1α (P = 0.002). **C.** The survival analysis of HIF-lα IHC results. PDAC patients (n = 158) with mild positive HIF-lα protein expression (−/+/++, n = 119) had significantly better total survival than those with strong positive expression (+++, n = 39). HIF-1α expression was prognostic for overall survival. The two- and three-year survival rates were 25.2% and 13.4%, respectively, in patients with mild HIF-1α, but decreased to 7.7% or 2.6%, respectively, in patients with strong expression of HIF-1α. (P<0.001) **D.** The survival analysis of CypA IHC results. The same PDAC patients (n = 158) with negative CypA protein expression (−, n = 65) had significantly better total survival than those with positive expression (+, n = 93). Patients with negative CypA had two- and three-year survival rates of 32.3% and 20%, respectively, which significantly decreased to 12.9% and 4.3% in patients with positive CypA (P<0.001). **E.** The survival analysis of HIF-lα and CypA IHC results. The same PDAC patients (n = 158) with mild positive HIF-1α and negative CypA protein expression (n = 57) had significantly better total survival than those with strong positive HIF-1α and positive CypA expression (n = 31), the two- or three-year survival rate between them was significantly different, respectively (P<0.001). Besides, the same PDAC patients (n = 158) with mild positive HIF-1α and positive CypA protein expression (n = 62) or with strong positive HIF-1α and negative CypA protein expression (n = 8) had significantly better total survival than those with strong positive HIF-1α and positive CypA expression (n = 31), and had worse total survival than those with mild positive HIF-1α and negative CypA expression (n = 57). The two- or three-year survival rate between the above every combination of two groups was significantly different, respectively (P<0.05).

To explore the function of HIF-1α and CypA expression in PDAC, we performed IHC to detect protein expression. Among 158 patients, our results showed that HIF-1α and CypA were positive in 122 and 124,respectively. CypA protein expression was significantly correlated with HIF-lα, as detected by immunohistochemical staining on PDAC (Figure.1A). Cross tabulation of HIF-lα and CypA expression levels in 158 cases of PDAC predicated that the expression of CypA was correlated with HIF-1α (P = 0.002). (Figure.1B).To analyze the relationship between CypA or HIF-1α protein levels and clinical factors, we compared the distribution with different clinical factors. During the follow-up period, all of the 158 patients and their relatives succumbed to their diseases. First, we found that HIF-1α expression had a role in prognosis: PDAC patients (n = 158) with mild positive HIF-lα protein expression (−/+/++, n = 119) had a significantly better total survival than those with strong positive expression (+++, n = 39). The two- and three-year survival rates were 25.2% and 13.4%, respectively, in patients with mild HIF-1α, but decreased to 7.7% or 2.6%, respectively, in patients with strong expression of HIF-1α. (P<0.001) (Figure.1C). Then our results indicated CypA expression also had a role in prognosis: The PDAC patients (n = 158) with negative CypA protein expression (−, n = 65) had a significantly better total survival than those with positive expression (+, n = 93). Patients with negative CypA had two- and three-year survival rates of 32.3% and 20%, respectively, which significantly decreased to 12.9% and 4.3% in patients with positive CypA (P<0.001) (Figure.1D).Additionally, there are cases with high level of HIF-1α. but low or no CypA. Similarly, very low level of or no HIF-1α. but positive CypA. Therefore, we added a survival plot that could consider both HIF-1α and CypA and categories low HiF-1α combined with high CypA, high HiF-1α combined with low CypA, both high and both low, which could provide better correlation of these two together as prognostic biomarkers: The PDAC patients (n = 158) with mild positive HIF-1α and negative CypA protein expression (n = 57) had a significantly better total survival than those with strong positive HIF-1α and positive CypA expression (n = 31), the two- or three-year survival rate between them was significantly different, respectively (P<0.001). (Figure.1E). Besides, the same PDAC patients (n = 158) with mild positive HIF-1α and positive CypA protein expression (n = 62) or with strong positive HIF-1α and negative CypA protein expression (n = 8) had significantly better total survival than those with strong positive HIF-1α and positive CypA expression (n = 31), and had worse total survival than those with mild positive HIF-1α and negative CypA expression (n = 57). The two- or three-year survival rate between the above every combination of two groups was significantly different, respectively (P<0.05: Figure.1E).

Table 1 showed both HIF-1α and CypA expression levels were significantly associated with lymph node metastasis (P = 0.012 and P = 0.007, respectively) and tumor stage (P = 0.032 and P<0.001, respectively).

These results indicated that CypA and HIF-1α were over-expressed in PDAC, and their expression levels could predict poor outcome.

### 2. CypA expression is regulated by HIF-1α

**Figure 2:**
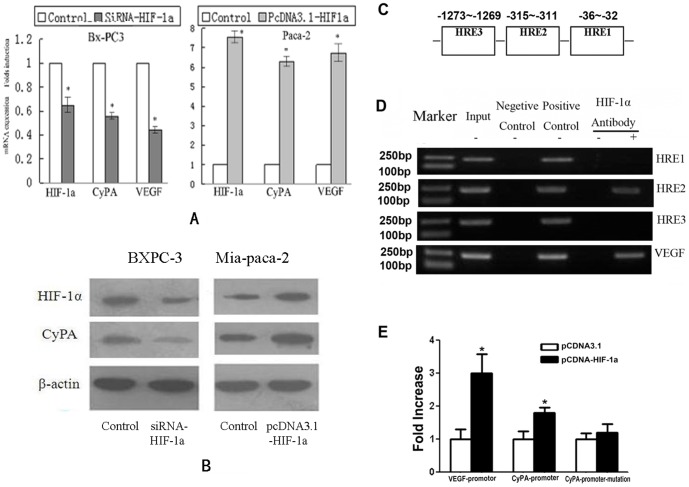
CypA expression is regulated by HIF-lα. **A.** HIF-lα and CypA relative mRNA expression levels in different groups. HIF-lα and CypA relative mRNA expression levels in pancreatic cancer cell lines Mia-paca-2 and BXPC3 were determined by real-time PCR analysis. β-actin cDNA served as the internal control. Expression levels in both untransfected cell lines were set as 1. Columns, mean of triplicate samples from two different experiments; bars, SE. *: P<0.05. **B.** HIF-lα and CypA protein expression levels in different groups. HIF-lα and CypA protein expression levels were determined by western blot analysis. β-actin served as the loading control. Expression levels in both untransfected cell lines were set as 1. Columns, mean of triplicate samples from two different experiments; bars, SE. *: P<0.05. **C.** Binding of HIF-lα to the CypA promoter in BXPC3.: screening of the 5-flanking region of the CypA gene revealed 3 potential HIF-1α binding sites (−32∼−36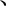 −311∼−315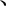 −1269∼−1273) relative to the start site of transcription. D. ChIP results in different groups. ChIP assay was performed and the promoter fragment binding sites (−311∼−315) was specifically co-immuno-precipitated by anti-HIF-lα but not the control IgG or non-antibody. In chromatin fraction pulled down by anti-HIF-1α antibody, the CypA promoter PCR fragments binding sites (−32∼−36 or −1269∼−127) hardly has been detected. E. Dual luciferase results in different groups. The results showed that the full-length CypA promoter activity (CypA-promotor) increased significantly after over expression of HIF-1α compared to that treated with plasmids alone (P<0.05). But the mutant CypA promoter (CypA-promotor-mutation) had not increased, which was used as negative control. VEGF luciferase reporter construct (VEGF-promotor) was used as positive control for the HIF-1α response. Over-expression of HIF-1α increased about 3-fold of the VEGF promoter activity compared to controls (P<0.05).

The relationship between CypA and HIF-1α was investigated. HIF-lα was highly expressed in BXPC3 cells, but not in Mia-paca-2 cells (Figure.2A). To determine the consequences of over-expression or suppression of HIF-lα in PDAC, siRNA HIF-1α interference was applied into pancreatic cancer cell line BXPC3 to inhibit HIF-lα expression, and pcDNA HIF-lα was applied into Mia -paca-2 cells to over-express HIF-lα. Real-time PCR and western blot analysis were used to detect the mRNA and protein expression levels of CypA and HIF-1α. After siRNA HIF-1α interference was applied into the BXPC3 cells, both the mRNA (P<0.05, respectively: Figure.2A) and protein expression levels (0.559 and 0.187, respectively: Figure.2B) of HIF-lα and CypA decreased. After pcDNA HIF-lα was applied into Mia Paca-2 cells, both the mRNA (P<0.05, respectively: Figure.2A) and protein (3.22 and 1.88, respectively: Figure.2B) expression levels of HIF-lα and CypA increased. These results indicated that the up-regulation or suppression of HIF-lα in PDAC promoted or suppressed the expression of CypA in vitro,respectively.

Then screening of the 5-flanking region of the CypA gene, we found 3 potential HIF-1α binding sites (−32∼−36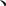 −311∼−315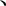 −1269∼−1273) relative to the start site of transcription (Figure.2C). To further investigate the relationship between CypA and HIF-1α in PDAC cells and to validate the binding of HIF-lα with the CypA promoter, an independent ChIP assay was performed and the promoter fragment binding sites (−311∼−315) was specifically co-immuno-precipitated by anti-HIF-lα but not the control IgG or non-antibody (Figure.2D). In chromatin fraction pulled down by anti-HIF-1α antibody, the CypA promoter PCR fragments binding sites−32∼−36 or −1269∼−1273) hardly has been detected(Figure.2D). Therefore, the CypA promoter region (−32∼−36 or −1269∼−1273) could not be pulled down by the anti-HIF-1α antibody, and HIF-1α could specifically bind to the HRE region (−311∼−315) of the CypA promoter. In order to determine whether the binding of HIF-1α to the CypA promoter could activate it, transient transfection and dual luciferase assay were performed to detect the CypA promoter activity with or without over-expression of HIF-1α. The results showed that the full-length CypA promoter activity (CypA-promotor) increased significantly after over expression of HIF-1α compared to that treated with plasmids alone (P<0.05: Figure.2E). But the mutant CypA promoter (CypA-promotor-mutation) did not increase, which was used as negative control. VEGF luciferase reporter construct (VEGF-promotor) was used as positive control for the HIF-1α response. Over-expression of HIF-1α increased about 3-fold of the VEGF promoter activity compared to controls (P<0.05: Figure.2E). Thus, HIF-1α could transactivate CypA expression.

### 3. Influence of HIF-1α and CypA on the biological behaviors of PDAC cell lines

To investigate the influence of HIF-1α and CypA on the biological behaviors of PDAC cell lines, siRNA-HIF-1α and/or siRNA-CypA were used to inhibit HIF-lα and CypA in BXPC3 cell lines, respectively. MTT was used to detect cell proliferation ability, and flow cytometry was used to detect the apoptotic changes. Scratch test and transwell slides were used to detect cell migration and invasion, respectively.

**Figure 3:**
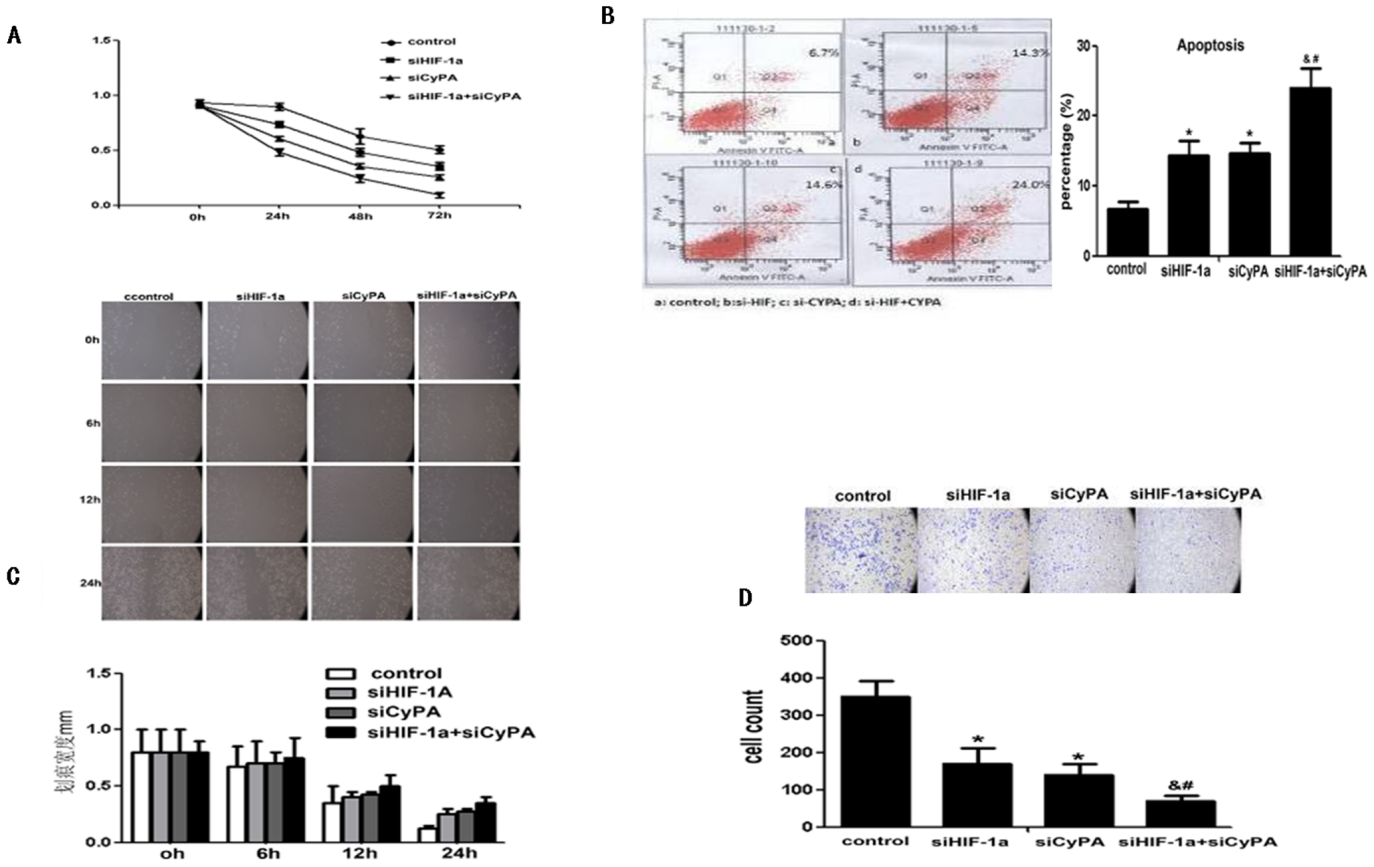
Influence of HIF-1α and CypA on the biological behavior of PDAC cell. **A.** MTT results showed the siRNA-HIF-1α and siRNA-CypA effect on cell proliferation. After transfection by siRNA-HIF-lα and/or siRNA-CypA in BXPC3 cells, cell proliferation was initially similar in the interference and control groups (P>0.05). The proliferative ability in the siRNA-HIF-lα and/or siRNA-CypA interference groups significantly decreased at 48 h to 72 h (P<0.05, respectively); and the combination of siRNA-HIF-lα and siRNA-CypA decreased proliferative ability compared to siRNA-HIF-lα or siRNA-CypA alone (P<0.05, respectively). **B.** FACS results showed the siRNA-HIF-1α and siRNA-CypA effect on cell apoptosis. After transfection by siRNA-HIF-lα and/or siRNA-CypA, cell apoptosis increased (P<0.05, respectively); and the combination of siRNA-HIF-lα and siRNA-CypA increased cell apoptosis compared to siRNA-HIF-lα or siRNA-CypA alone (P<0.05, respectively). **C.** The scratch results showed the siRNA-HIF-1α and siRNA—CypA effect on cell migaration. The scratch space at 0, 6, 12, and 24 h of BXPC3 cells was detected after transfection by siRNA-HIF-lα and/or siRNA-CypA: The widths in the transfer groups were bigger than those in the control group at 12 and 24 h (P<0.05, respectively); and the combination of siRNA-HIF-lα and siRNA-CypA increased scratch space compared to siRNA-HIF-lα or siRNA-CypA alone (P<0.05, respectively). **D.** The transwell slide results showed the siRNA-HIF-1α and siRNA—CypA effect on cell invation. After transfection by siRNA-HIF-lα and/or siRNA-CypA, the transwell results showed the cell number through the membrane was significantly less than the control group (P<0.05); and the combination of siRNA-HIF-lα and siRNA-CypA decreased the cell number through the membrane compared to siRNA-HIF-lα or siRNA-CypA alone (P<0.05, respectively).

Firstly, after transfection by siRNA-HIF-lα and/or siRNA-CypA in BXPC3 cells, cell proliferation was initially similar in the interference and control groups (P>0.05: Figure.3A). The proliferative ability in the siRNA-HIF-lα and/or siRNA-CypA interference groups significantly decreased at 48 h to 72 h (P<0.05, respectively: Figure.3A); and the combination of siRNA-HIF-lα and siRNA-CypA decreased proliferative ability compared to siRNA-HIF-lα or siRNA-CypA alone (P<0.05,respectively: Figure.3A). Secondly, after transfection by siRNA-HIF-lα and/or siRNA-CypA, cell apoptosis increased (P<0.05, respectively: Figure.3B); and the combination of siRNA-HIF-lα and siRNA-CypA increased cell apoptosis compared to siRNA-HIF-lα or siRNA-CypA alone (P<0.05, respectively: Figure.3B). Thirdly, the scratch space at 0, 6, 12, and 24 h of BXPC3 cells was detected after transfection by siRNA-HIF-lα and/or siRNA-CypA: The widths in the transfer groups were bigger than those in the control group at 12 and 24 h (P<0.05, respectively: Figure.3C); and the combination of siRNA-HIF-lα and siRNA-CypA increased scratch space compared to siRNA-HIF-lα or siRNA-CypA alone (P<0.05, respectively: Figure.3C). And lastly, a.nd after transfection by siRNA-HIF-lα and/or siRNA-CypA,CypA, the transwell results showed the cell number through the membrane was significantly less than the control group (P<0.05: Figure.3D); and the combination of siRNA-HIF-lα and siRNA-CypA decreased the cell number through the membrane compared to siRNA-HIF-lα or siRNA-CypA alone (P<0.05, respectively: Figure.3D).

These results indicated that HIF-1α and/or CypA could promote PDAC cell proliferation, migaration and invasion, but suppress its apoptosis in vitro.

### 4. Synergistic effect of CsA and 2ME against PDAC in vitro

To prove the effects of HIF-1α and CypA in PDAC, the combined anti-cancer effect of 2ME and CsA in PDAC was tested in vitro.

**Figure 4:**
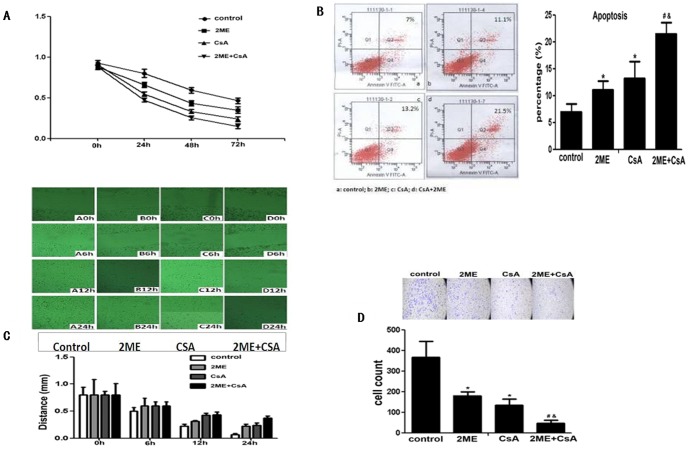
Combined anti-cancer effect of 2ME and CsA in pancreatic cancers in vitro. **A.** MTT results showed the 2ME and CsA effect on cell proliferation. After treated by 2ME and/or CsA in BXPC3 cells, cell proliferation was initially similar in the interference and control groups (P>0.05). The proliferative ability in the 2ME and/or CsA treated groups significantly decreased at 48 h to 72 h compared to the control group (P<0.05, respectively: Figure. 4A); and the combination of 2ME and CsA decreased proliferative ability compared to 2ME or CsA alone (P<0.05, respectively). **B.** FACS results showed the 2ME and CsA effect on cell apoptosis. After treated by 2ME and/or CsA in BXPC3 cells, cell apoptosis was increased compared to the control group (P<0.05, respectively); and the combination of 2ME and CsA increased cell apoptosis compared to 2ME or CsA alone (P<0.05, respectively). **C.** The scratch results showed the 2ME and CsA effect on cell migaration. The scratch space at 0, 6, 12, and 24 h of BXPC3 cells was detected after treated by 2ME and/or CsA in BXPC3 cells: the widths in the 2ME and/or CsA treated groups were bigger than those in the control group at 12 and 24 h (P<0.05, respectively); and the combination of 2ME and CsA increased scratch space compared to 2ME or CsA alone (P<0.05, respectively). **D.** The transwell slide results showed the 2ME and CsA effect on cell invation. After treated by 2ME and/or CsA in BXPC3 cells, the transwell results showed the cell number through the membrane was significantly less than the control group (P<0.05); and the combination of 2ME and CsA decreased the cell number through the membrane compared to 2ME or CsA alone (P<0.05, respectively).

First, after treated by 2ME and/or CsA in BXPC3 cells, cell proliferation was initially similar in the interference and control groups (P>0.05: Figure.4A). The proliferative ability in the 2ME and/or CsA treated groups significantly decreased at 48 h to 72 h compared to the control group (P<0.05, respectively: Figure. 4A); and the combination of 2ME and CsA proliferative ability decreased compared to 2ME or CsA alone (P<0.05, respectively: Figure.4A). Second, after treated by 2ME and/or CsA in BXPC3 cells, cell apoptosis was increased compared to the control group (P<0.05, respectively: Figure. 4B); and the combination of 2ME and CsA increased cell apoptosis compared to 2ME or CsA alone (P<0.05, respectively: Figure.4B). Third, the scratch space at 0, 6, 12, and 24 h of BXPC3 cells was measured after treated by 2ME and/or CsA in BXPC3 cells: the widths in the 2ME and/or CsA treated groups were bigger than those in the control group at 12 and 24 h (P<0.05, respectively: Figure.4C); and the combination of 2ME and CsA increased scratch space compared to 2ME or CsA alone (P<0.05, respectively: Figure.4C). At last, after treated by 2ME and/or CsA in BXPC3 cells, the transwell results showed that the cell number through the membrane was significantly less than that in the control group (P<0.05: Figure.4D); and that the number of the cell coming through the membrane was decreased in the combination group of 2ME and CsA compared to 2ME or CsA alone (P<0.05, respectively: Figure.4D).

These results indicated the combined suppression effect of 2ME and CsA in vitro: 2ME and/or CsA could promote PDAC cell proliferation, migaration and invasion, but suppress its apoptosis in vitro.

### 5. Synergistic effect of CsA and 2ME against PDAC in vivo

**Figure 5:**
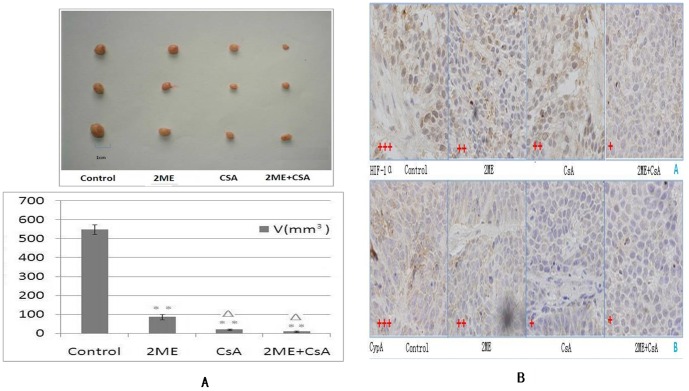
Combined anti-cancer effect of 2ME and CsA in pancreatic cancers in vivo. **A.** The mice tumors' volume in different groups. After six weeks of treatment, 2ME and/or CsA therapy decreased the tumors' volume, which was significantly smaller than those in the PBS control group (P<0.05, respectively); and the combination therapy decreased the tumor volume to 22.22±4.38 mm^3^, which was significantly smaller than those in the PBS and single-agent therapies (P<0.001, respectively). **B.** IHC results in different groups. Additionally, the IHC results confirmed the effect of 2ME and/or CsA: 2ME and/or CsA therapy decreased the HIF-lα and/or CypA positive tumor cells compared to those in the PBS control group; and the combination therapy decreased the positive tumor cells compared to PBS or single-agent therapies.

We further investigated the anti-cancer effect of 2ME and CsA on pancreatic cancer in vivo. Approximately 2×10^7^ BXPC3 cells were injected subcutaneously into 12 SCID mice (four to six weeks old, with body weight of 14 g to 16 g), and the tumor volume was measured. After three weeks of injection, all mice had a tumor volume of 64.60±3.80 mm^3^. The mice were randomized into four groups, with mean tumor volumes of 64.32±8.48, 62.96±6.99, 62.13±8.26, and 68.00±8.02 mm^3^. After randomization, the mice were treated as detailed in the Materials and Methods. After six weeks of treatment, 2ME and/or CsA therapy decreased the tumors' volume, which was significantly smaller than those in the PBS control group (P<0.05, respectively: Figure.5A); and the combination therapy decreased the tumor volume to 22.22±4.38 mm^3^, which was significantly smaller than those in the PBS and single-agent therapies (P<0.001, respectively: Figure.5A). Additionally, the IHC results confirmed the effect of 2ME and/or CsA: 2ME and/or CsA therapy decreased the HIF-lα and/or CypA positive tumor cells compared to those in the PBS control group; and the combination therapy decreased the positive tumor cells compared to compared to those in the PBS and single-agent therapies (Figure.5B). Treatment with 2ME and/or CsA resulted in no significant difference in the body weight of treated mice. None of the tested mice manifested signs of other adverse effects as specified in the Method section. No toxicity on the blood count or hepatic and renal function was observed with doses of 2ME alone, CsA alone, or their combination (data not shown). These results indicated the anti-tumor effect and safety of 2ME and/or CsA in vivo.

## Discussion

This study indicated that 77.2% (122/158) of the investigated PDAC samples demonstrated (+/++/+++)HIF-1 expression, which was consistent with previous reports [10], [21]. CypA expression was positive in 78.5% (124/158) of the samples. Besides, our results indicated the expression levels of CypA and HIF-1α were over-expressed in PDAC, and their could predict poor outcome. Therefore, both of the two protein could be considered as prognostic factors associated with poorer prognosis. A correlation between their expression levels was observed in PDAC tissues.

Next, we studied the relationship between HIF-1α and CypA in vitro. The mRNA and protein expression levels of CypA markedly decreased or increased following the suppression or over-expression of HIF-1α in PDAC cell lines, respectively. The expression of CypA was similar to that of VEGF, which is one of the HIF-1α downstream genes. Besides, we found 3 potential HIF-1α binding sites relative to the start site of transcription. To identify the downstream target genes of the HIF-1α, we performed ChIP-on-chip analysis and found that CypA was a target gene of HIF-1α (unpublished data). Furthermore, to validate the binding of HIF-1α with CypA promoter, through the binding of HIF-1α to the CypA promoter in pancreatic cancer cell line BXPC3, an independent ChIP assay was performed and the promoter fragment was specifically co-immunoprecipitate by anti- HIF-1α, so we proved that HIF-1α could bind directly to the hormone response element sites in the CypA promoter area and regulate CypA expression.

Then we investigated the influence of HIF-1α and CypA expression levels on cell proliferation, apoptosis, and invasion in vitro. Consistent with other studies [4]–[10], [13]–[18], HIF-1α and CypA promoted PDAC cell proliferation, invasion and migration, and suppressed PDAC cell apoptosis in vitro.

The expression of HIF-1α might be activated by AKT phosphorylation, as we previously reported [6], whereas the over-expression of CypA might stimulate the proliferation of human pancreatic cancer cells by activating the ERK1/2 and p38 pathways [27]–[30]. But CypA expression is not solely under regulation of HIF-1α, and cholecystokinin (CCK), p532 could regulate CyPA, which could regulate IκB-α, NF-Κb p65, CD147, MMP9 and so on [13]–[20]. Therefore, the relationship between HIF-1α and CypA warrants further study: Increased CypA levels could reverse the inhibitory effect of decreased HIF-1α on PDAC cell behavior, but increased HIF-1α could not reverse the inhibitory effect of decreased CypA on PDAC cell behavior.

Cross-talk between different signal pathways may partly explain the poor response rate of HIF-1α and CypA inhibitors as single-agent therapy in solid tumors [27]–[30]. Thus, we analyzed the combination therapy of 2ME and CsA in PDAC in vitro and in vivo. We demonstrated a synergistic anti-cancer effect of the two agents.

CsA inhibits the PPIase activity of CypA and chemotherapy resistance induced by P-glycoprotein, and induces apoptosis in cancer cells [31], [32]. However, the immunosuppressive effect and the appearance of de novo cancers hinder the application of CsA in cancer treatment [33], [34]. Recent studies reported that CsA might enhance the chemotherapeutic effect of cytotoxic agents in various cancers [20], [31], [32], [34], whereas HIF-1α knockdown might increase chemo-sensitivity in pancreatic cancer cell line [35]. In our study, the synergistic anti-cancer effect of 2ME/CsA was analyzed. In vivo analysis indicated that combination therapy had no toxicity on blood count and liver/renal function, which was not agreed with previous reports [36]–[38]. With evidence that long-term use of CsA might have a positive function in cancer prevention [39], we only provided preliminary data for further clinical trials on the combination therapy of 2ME/CsA.

As previously reported, CypA can desensitize various cancer cells to hypoxia- or cisplatin-induced cell death by the suppression of ROS [24]. The accumulation of ROS activates the AKT pathway via the suppression of PTEN and activation of NF-κB [25], [26]. The activation of AKT reportedly stimulates HIF-1α synthesis [27]–[29], which might eventually activate downstream genes to eliminate extra ROS. Thus, we hypothesized that CsA might partly induce cell death as a result of ROS accumulation, and that 2ME might enhance the intracellular ROS accumulation.HIF-1α can inhibit mitochondrial respiration and subsequently decrease intracellular ROS generation [40]. This decrease was reversed by 2ME in vitro, because AKT activation is accompanied by the suppression of forkhead transcription factors (FOXOs), a key factor in ROS degradation[41].

Another mechanism of the synergistic effect of 2ME/CsA might be the disruption of the microtubule structure. 2ME could bind to tubulin dimers and cause the depolymerization of microtubules, which could initiate the signaling pathways of apoptosis [11], [12], [42], [43]. And the reports indicated that microtubule-disrupting agents might cause dramatic subcellular redistribution of CypA from the nucleus to the cytosol and plasma membrane, which might be an important part of the mechanism by which microtubule-disrupting agents exert their effects [42]–[44].

In summary, our results indicated that HIF-1α and CypA expression were prognostic factors in PDAC. A correlation was observed between the expression of the two hypoxia-associated factors in primary PDAC tissues. HIF-1α promoted the development of PDAC by transactivating CypA, which might serve as a potential therapeutic target for PDAC. Furthermore, HIF-1α and CypA promoted PDAC cell proliferation, migaration and invasion, and suppressed its apoptosis in vitro. Finally, we demonstrated the synergistic anti-tumoreffect by the combination of 2ME and CsA in PaCa-2 cell line in vitro and in vivo. Furthermore the synergistic anticancer effect of 2ME and CsA warrants further confirmation in clinical trials.
